# Intra-individual variability and circadian rhythm of vascular endothelial growth factors in subjects with normal glucose tolerance and type 2 diabetes

**DOI:** 10.1371/journal.pone.0184234

**Published:** 2017-10-09

**Authors:** Markolf Hanefeld, Katrin Engelmann, Dieter Appelt, Dirk Sandner, Ingo Weigmann, Xenia Ganz, Frank Pistrosch, Carsta Köhler, Antje Gasparic, Andreas L. Birkenfeld

**Affiliations:** 1 Study Centre Metabolic Vascular Medicine, GWT TU-Dresden GmbH, Dresden, Germany; 2 Medical Clinic III, Universitätsklinikum "Carl Gustav Carus", Technische Universität Dresden, Dresden, Germany; 3 Klinikum Chemnitz gGmbH, Chemnitz, Germany; 4 Experimental Ophthalmology, Institute of Anatomy, Technische Universität Dresden, Dresden, Germany; 5 Clinic for Ophthalmology, Universitätsklinikum "Carl Gustav Carus", Technische Universität Dresden, Dresden, Germany; 6 Medical Consulting, GWT TU-Dresden GmbH, Dresden, Germany; 7 Paul Langerhans Institute Dresden of the Helmholtz Center Munich at University Hospital and Faculty of Medicine, TU Dresden, Dresden, Germany; 8 German Center for Diabetes Research (DZD e.V.), Neuherberg, Germany; 9 Section of Diabetes and Nutritional Sciences, Rayne Institute, Denmark Hill Campus, King’s College London, United Kingdom; Medical Clinic, University Hospital Tuebingen, GERMANY

## Abstract

Increased levels of systemic vascular endothelial growth factors (VEGFs) in patients with diabetes are associated with increased risk of microvessel disease. On the other hand, low VEGF levels after intravitreal antibody application may be associated with acute cardiovascular complications and treatment failure. Individual levels of systemic VEGF vary in a wide range depending on analytical methods and quality of diabetes control. So far only limited information exists on intraindividual fluctuations over longer periods and circadian rhythms. We analysed the intraindividual variance of VEGF-A, VEGF-C and placental growth factor (PLGF) in CTAD (citrate-theophylline-adenine-dipyridamol) plasma as well as VEGF-A in serum over a period of 6 months in patients with stable controlled type 2 diabetes (10 M, 10 F) and age and sex matched subjects with normal glucose tolerance (NGT). Furthermore, circadian levels of VEGFs were controlled hourly from 7:30 a.m. to 7:30 p.m. under standardized metabolic ward conditions. In addition, the relationship to metabolic, hormonal and inflammatory biomarkers was analyzed. VEGF-A, VEGF-C and PLGF remained stable in plasma and VEGF-A in serum over 6 months in both groups. No circadian change was observed in VEGF-A serum and plasma concentrations. A minor decrease of VEGF-C plasma levels was evident after 5 p.m. in both groups and a significant peak of PLGF concentrations occurred after lunch, which was more pronounced in T2DM. In multivariate analysis, only serum VEGF-A correlated to diabetes duration, whereas VEGF-C only correlated to HbA1c and fasting blood glucose. We did not observe significant intraindividual variances for VEGF-A in serum and VEGF-A, VEGF-C and PLGF in CTAD plasma over a period of 6 months. Taken together, a single morning measurement of systemic VEGF levels after 7:30 am appears to be a reliable parameter for the individual risk associated with abnormal VEGF concentrations in blood.

Trial Registration: NCT02325271

## Introduction

Vascular endothelial growth factors (VEGF) are important regulators of endothelial function, regulating blood-retinal barrier, filtration of macromolecules in the kidney and neo-angiogenesis in endorgans in the case of hypoxia [1,2]. Clinical studies in patients with diabetes have established a pivotal role of VEGF in the incidence and progression of diabetic retinopathy (DR) [3,4]. This has led to the development of specific anti VEGF antibodies as a successful intravitreal treatment of proliferative retinopathy and diabetic macular edema (DME) [3,5,6]. Since anti-VEGF therapy is available for the treatment of age related macular degeneration (ARMD) and DME, the high incidence of blindness could be reduced [7]. However, it is still matter of debate how the anti-VEGF therapy can be best guided. A systematic screening of potential biomarkes of the diabetic population with and without retinal disorders seems to be inevitable for an optimal guidance of the patients through the long lasting therapy.

Although cross sectional studies have demonstrated increased levels of VEGF, mostly VEGF-A, in serum and plasma of patients with proliferative diabetic retinopathy and DME, the contribution of systemic VEGF to DR and diabetic nephropathy remains unclear. VEGF-A is a member of the VEGF family that includes VEGF-B, VEGF-C, VEGF-D, VEGF-E and placental growth factor (PLGF). Individual levels in cohorts without diabetes and even more with diabetes vary in a wide range depending on quality of diabetes control, biometric factors, medications, such as statins, individual activity of low grade inflammation and preanalytical handling [8–13]. Main sources of VEGF in serum are platelets and leucocytes [14], which are activated by chronic hyperglycemia [15]. Therefore, many studies use plasma with different platelet inhibitors [8,16–18] but even this is associated with great interindividual differences in well characterized patients with DR [8]. In previous studies we could show that VEGF-A levels measured in EDTA plasma were in the same range for patients with and without DME [8].

So far, it remains an open question whether circulating individual VEGF levels are valid risk factors or risk markers for both, development and progression of DR and DME. Moreover, there are still many open questions which methods should be used and which VEGF fractions are the best indicators of risk or as risk markers. Therefore, this study specifically addressed two questions:

1. Intraindividual variation of different VEGF fractions in serum and plasma of patients with type 2 diabetes under stable conditions and controls with normal glucose tolerance
2. Circadian fluctuations of VEGF in these patients.

### Study population and methods

We analyzed 20 patients with type 2 diabetes and stable glucose control (10 male, 10 female) and 20 age and sex matched subjects with normal glucose tolerance. Normal glucose tolerance was verified by a 75g oral glucose tolerance test. Inclusion criteria for patients with diabetes were: Age 40–80 years, diabetes duration 5 to 25 years, HbA1c between 6.5% and 9%, informed consent. Exclusions were diabetic retinopathy, hsCRP ≥ 10 mg/l, acute infections within 14 days prior to entry or during the follow up, acute or chronic inflammatory diseases, treatment with anti-inflammatory or immunosuppressive drugs, acute myocardial infarction or coronary syndrome or stroke within 1 year before the study, cancer or cancer treatment within 5 years prior to study inclusion.

**Fig 1:**
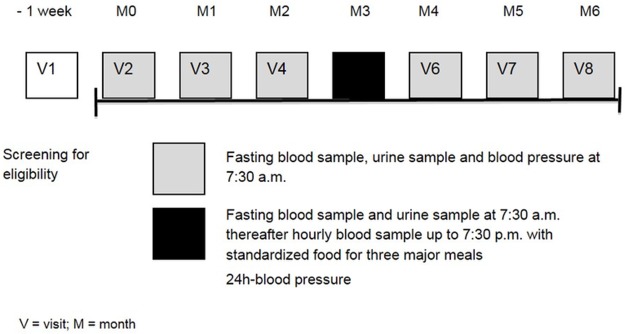
Study design.

Intraindividual variability was measured over 6 months (Fig 1) and circadian fluctuations of primary target parameters at visit 5 under standardized conditions in our outpatient department. Blood samples for primary and secondary laboratory parameters were taken after overnight fasting between 7.30 and 8.00 at morning. Additionally vital signs and ambulatory arterial blood pressure measured combined with a medical history. Circadian rhythm was analyzed by measuring different parameters at visit 5, hourly between 7.30 AM and 7.30 PM under standardized ward conditions with three body weight adjusted calory-standardized meals (8.00 AM, 12.30 PM and 6.30 PM). As the major member of the growth factor family, VEGF-A was measured in serum and CTAD plasma using ELISA (IBL International GmbH, Ref. BE55101, Hamburg, Germany). VEGF-C (IBL International GmbH, Ref. BE55111, Hamburg, Germany) and PLGF (IBL International GmbH, Ref. BE52361, Hamburg, Germany) were analysed in plasma, VEGF-B had to be excluded because of analytical problems with the test kit. HbA1c was measured with high performance liquid chromatography, glucose was determined enzymatically using an UV method utilizing hexokinase. Creatinine was measured by enzymatic-colorimetric testing (PAP) and glomerular filtration rate (GFR) was calculated using the MDRD-formula (eGFR [ml/min/1,73m2] = 186 x (serum-crea[mg/dl])-1,154 x (age[years])-0,203 x (0,742 if female).

Metalomatrix proteinase-9 (MMP-9) (Asbach Medical Products GmbH, LOT-Nr. AMP60-E281300, Obrigheim, Germany), adiponectin (Asbach Medical Products GmbH, LOT-Nr. AMP 40-E24000, Obrigheim, Germany), VEGF-A, VEGF-C, PLGF, glucose, HbA1c, creatinine and eGFR levels were analysed for 6 months with standardized monthly measurements in the morning in the fasted state. These measurements were repeated hourly for 12 hours at visit 5 as well as 24h-blood pressure monitoring once at visit 5 as described [19]. Data are available from Figshare at: https://figshare.com/s/b5fa09f1586ec3982308

### Statistical methods and sample size calculation

This descriptive exploratory study did not rely on formal statistical sample size estimation, since these data were lacking so far in patients with and without diabetes. Based on experience from previous VEGF-A related studies [9,11] the chosen sample size of 40 valid subjects (20 per dose group) was considered to be sufficient to fulfill the objectives of the study. Primary variables analyzed in the present study were the variance (CV) of systemic VEGF levels (-A, -C and PIGF) over time for 6 months with monthly measurements once daily in the morning in the fasted state and the circadian rhythm of systemic VEGF levels analyzed hourly over 12 hours at visit 5. Secondary parameters evaluated in this study included blood pressure, albumin, creatinine, HbA1c, glucose over time for 6 months with monthly measurements once daily in the morning and glucose and creatinine and blood pressure measured hourly for 12 hours at visit 5 as well as 24h-blood pressure monitoring once at visit 5.

Statistical analyses were performed using SPSS version 21. Quantitative variables were summarized using descriptive statistics (number of data points, arithmetic mean, standard deviation, median and 95% confidence interval). Qualitative variables were summarized using frequency tables. All 40 subjects who were allocated to the study groups had complete data of primary and secondary target variables at all study visits and no major deviations from the protocol. Thus, all probands were included in the per protocol analysis set. All statistical analyses were explorative. A possible relationship between primary and secondary parameters was tested by Pearson correlation analyses. To prove the variances of both groups, the Levene test was performed. The coefficient of variation (CV) was calculated to compare the standard deviation (SD) dependent on the arithmetic mean of two distributions. CV <1 was considered as low variance, whereas CV >1 means was considered as high variance.

## Results

Number of included patients is given in S1 File. As shown in Table 1, controls and type 2 diabetic patients were well matched for age, sex and arterial blood pressure. However diabetic patients had a significantly higher BMI, greater waist circumference, received more often statins and had higher triglycerides. The mean HbA1c was 5.58%±0.29% for subjects of the NGT group and 7.07%±0.49%in patients with diabetes.

**Table 1:** Demographic and baseline characteristics by study group.

Parameter	NGT N = 20 (100%)	T2DM N = 20 (100%)
**Gender** (n (%))		
Male	10 (50%)	10 (50%)
Female	10 (50%)	10 (50%)
**Age** (years)		
Mean / SD	66.1 / 6.21	68.0 / 4.70
95% CI	63.2–69.0	65.8–70.2
**BMI** (kg/m^2^)		
Mean / SD	26.3 / 3.35	29.1 / 3.71
95% CI	24.8–27.3	27.3–30.8
**HbA1c** (%)		
Mean / SD	5.58 / 0.29	7.07 / 0.49
95% CI	5.44–5.71	6.84–7.29
**Triglycerides** (mmol/L)		
Mean / SD	1.11 / 0.5	2.0 / 1.49
95% CI	0.88–1.35	1.30–2.70
**Waist circumference** (cm)		
Mean / SD	93.9 / 12.9^a^	102.0 / 10.0^a^
**Mean systolic blood pressure**^b^ (mmHg)		
Mean / SD	140 / 16.1	139 / 17.8
95% CI	133–148	131–148
**Mean diastolic blood pressure**^b^ (mmHg)		
Mean / SD	77.2 / 8.47	79.3 / 13.0
95% CI	73.2–81.1	73.2–85.4
**Statins (n (%))**	5 (25%)	10 (50%)
Simvastatin	3 (15%)	8 (40%)
Atorvastatin	2 (10%)	1 (5%)
Fluvastatin	0	1 (5%)

NGT: subjects with normal glucose tolerance, T2DM: patients with type 2 diabetes, SD: standard deviation, CI: confidence interval

^a^ n = 16

^b^ mean value according to measurement of blood pressure at left and right arm

**Fig 2:**
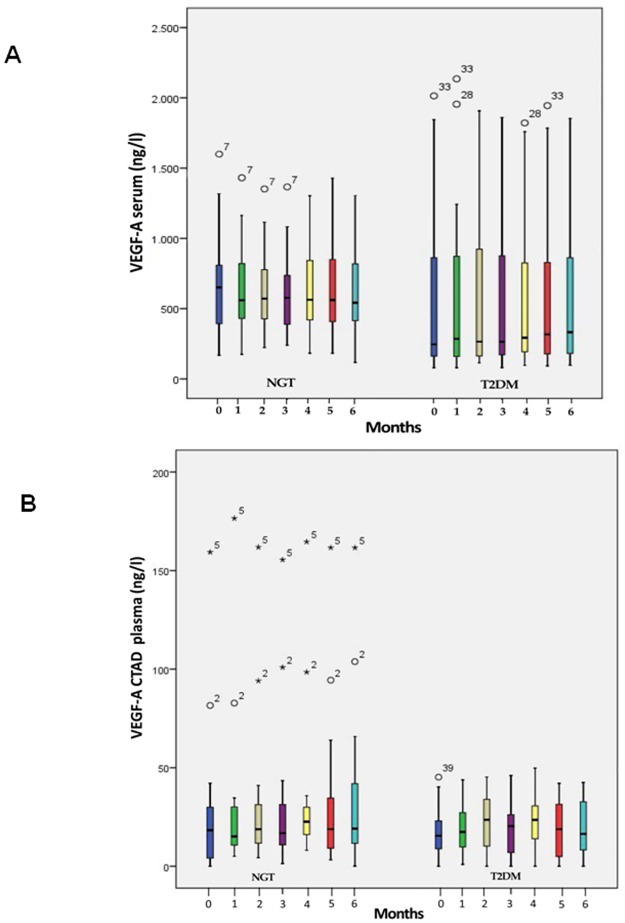
A) Boxplot of VEGF-A concentrations in serum of subjects with normal glucose tolerance (NGT) and patients with type 2 diabetes mellitus (T2DM) over an observation period of 6 months. B) Boxplot of VEGF-A concentrations in CTAD plasma of subjects with normal glucose tolerance (NGT) and patients with type 2 diabetes mellitus (T2DM) over an observation period of 6 months. The lower border of the box plots represent the 25th percentile and the upper border represents the 75th percentile ± SD. ° = patient with a value < 3 times box size, * = patient with a value ≥ 3 times box size. Number above °/* = reflects individual patient number.

**Fig 3:**
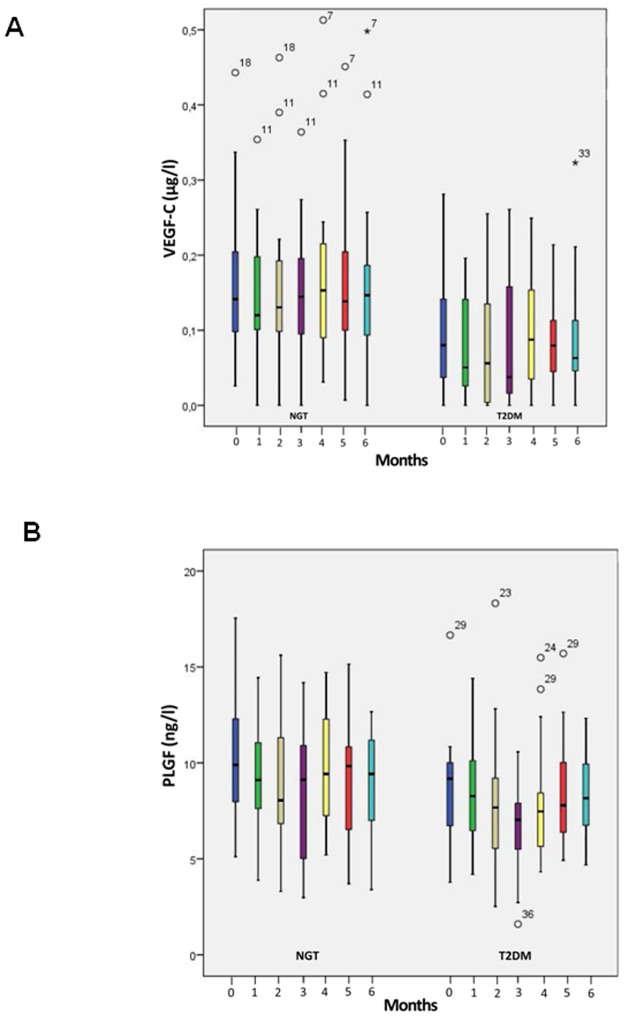
A) Boxplots of VEGF-C concentrations in plasma of subjects with normal glucose tolerance (NGT) and patients with type 2 diabetes mellitus (T2DM) over an observation period of 6 months B) Boxplots of PLGF concentrations in plasma of subjects with normal glucose tolerance (NGT) and patients with type 2 diabetes mellitus (T2DM) over an observation period of 6 months. The lower border of the box plots represent the 25th percentile and the upper border represents the 75th percentile ± SD. ° = patient with a value < 3 times box size, * = patient with a value ≥ 3 times box size. Number above °/* = reflects individual patient number.

Intraindividual levels of serum VEGF-A remained stable over a period of 6 months (Fig 2a) but with a wide interindividual range for both controls and patients with type 2 diabetes. Despite a baseline HbA1c level of 7.07 ± 0.49% VEGF-A was more scattered in subjects with type 2 diabetes as demonstrated in the box plots. Mean VEGF-A serum levels were lower in patients with type 2 diabetes (mean over 6 months: 554–588 ng/l) than controls (602–658 ng/l). The same characteristics were found for VEGF-A levels in CTAD plasma (mean T2DM group: 16.5–22.6 ng/l; NGT group: 26.4 μg/l—33.6 ng/l) (Fig 2b). Moreover, plasma levels for patients with diabetes as well as controls were magnitudes lower than those in serum. Also, plasma levels of VEGF-C (Fig 3a) and PLGF (Fig 3b) remained stable over the observation period of 6 months. In parallel with VEGF-A we observed a wide interindividual range for both fractions which was similar for controls and diabetic patients.

**Fig 4:**
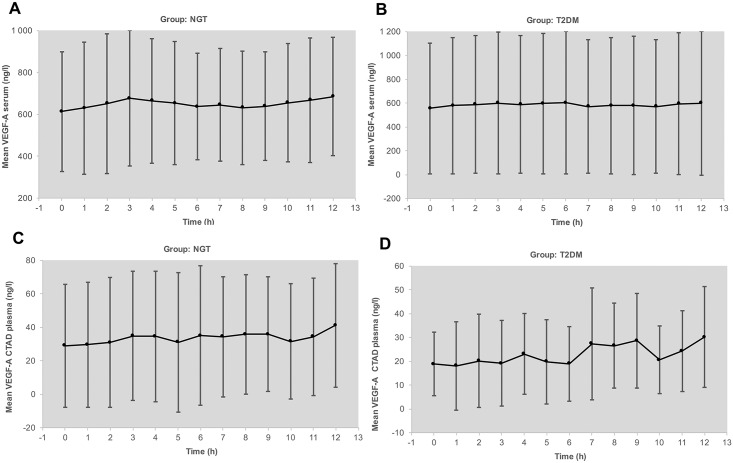
A) Circadian rhythm of VEGF-A levels (arithmetic mean and SD) in serum of subjects with normal glucose tolerance (NGT). B) Circadian rhythm of VEGF-A levels (arithmetic mean and SD) in serum of patients with type 2 diabetes mellitus (T2DM). C). Circadian rhythm of VEGF-A levels (arithmetic mean and SD) in plasma of subjects with normal glucose tolerance (NGT). D). Circadian rhythm of VEGF-A levels (arithmetic mean and SD) in plasma of patients with type 2 diabetes mellitus (T2DM).

**Fig 5:**
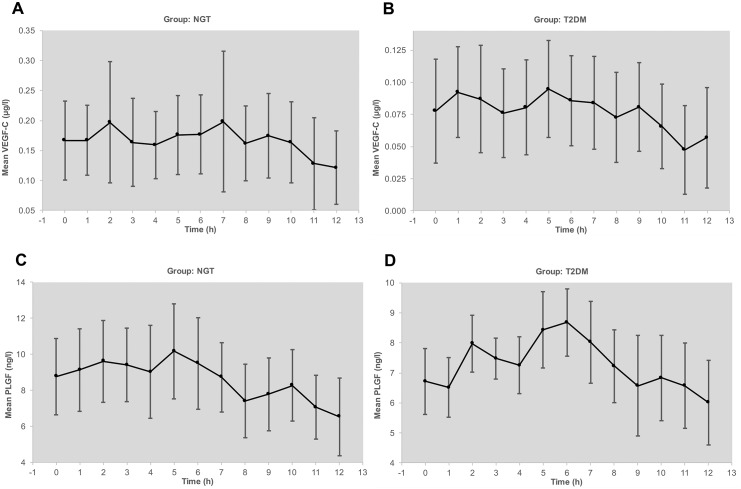
A) Circadian rhythm of VEGF-C levels (arithmetic mean and 95% CI) in plasma of subjects with normal glucose tolerance (NGT). B). Circadian rhythm of VEGF-C levels (arithmetic mean and SD) in plasma of patients with type 2 diabetes mellitus (T2DM). C). Circadian rhythm of PLGF levels (arithmetic mean and SD) in plasma of subjects with normal glucose tolerance (NGT). D) Circadian rhythm of PLGF levels (arithmetic mean and SD) in plasma of patients with type 2 diabetes mellitus (T2DM).

Circadian levels of VEGF-A serum and plasma remained at the same range between fasting at 7.30 AM and postprandial up to 7.30 PM (Fig 4a–4d) in both groups. However, we observed a distinct increase in postprandial levels of VEGF-C and PLGF (Fig 5a–5d). Parallel measurements of intraindividual levels of hsCRP and MMP-9 as biomarkers of low grade inflammation revealed minor variation under stable conditions. In contrast to VEGF, both biomarkers were higher in diabetic patients compared to controls. HsCRP as well as MMP-9 exhibited, however, no circadian fluctuations. Similarly, there were no substantial fluctuations over the observation period of 6 months for adiponectin, serum creatinine, and albumin/creatinine ratio in urine (Data are available from Figshare at: https://figshare.com/s/b5fa09f1586ec3982308). HbA1c was stable without any change in drug treatment and well controlled in the patients with diabetes at a mean level of 7.1%. Weight also remained unchanged.

Multivariate analysis of demographic data with VEGF as dependent variable revealed a positive correlation of serum VEGF-A (r = 0.50, p = 0.025) with the duration of diabetes only. Correlation analysis between biomarkers of inflammation and metabolic parameters with VEGF revealed a positive correlation of HbA1c and fasting glucose to VEGF-C.

## Discussion

This study analyzed intraindividual variances of VEGF-A in serum and plasma, as well as VEGF-C and PLGF in plasma under standardized conditions in two well characterized age and sex matched cohorts with and without diabetes over a period of 6 months. Our data show that all three VEGF fractions were stable in individuals under real world conditions controlled for possible biases. However, in both cohorts we observed a wide interindividual variation of VEGF concentrations in serum and plasma, which was more pronounced in patients with diabetes. This was valid for VEGF-A in plasma and serum. This data suggest that point measurements are valid indicators of a possible risk associated with an individual VEGF concentration in serum or plasma. Furthermore, no significant circadian fluctuation was observed for VEGF-A in serum and plasma in our study. Previous studies in patients with rheumatoid arthritis and healthy controls observed a slight increase in VEGF-A concentrations after 7 AM [11]. Since we did not sample VEGF-A before 7 am, we conclude that VEGF-A measurement can be securely and reliably sampled in the fasted or postprandial state after 7 AM. Previous studies also reported episodic fluctuations in VEGF-A levels in women with ovulation induction with human choriogonadotropin [20]. Women in our study were postmenopausal and we did not observe a sexual dimorphism in regard to VEGF-A plasma or serum levels.

In contrast to VEGF-A, circadian levels of VEGF-C and PLGF showed a tendency to higher levels in the postprandial phase after lunch and dinner. These changes occurred in parallel to changes in blood glucose excursions. Interestingly, VEGF-C was shown to be regulated by a synergistic action of VEGF-A and glucose in human retinal pigment epithelial cells [21]. Moreover, sodium chloride has recently been shown to increase the expression of VEGF-C in mononuclear phagocyte system cells [22]. Whether or not this mechanism is responsible for the postprandial effects observed in our setting deserves further study. So far, no conclusive data has been published providing convincing evidence for regulators of PLGF in humans *in vivo*.

In our exploratory study comparing patients with type 2 diabetes to control subjects matched for age and sex, VEGF-A levels were considerably lower in patients with HbA1c in target (7%) and without DR. This confirms previous studies showing that well-controlled diabetes is not associated with increased levels of VEGF [23,24]. Moreover, others and we have shown previously that serum VEGF-A increases with increasing HbA1c [8,11]. We did not observe correlations between VEGF-A, VEGF-C or PLGF and established biomarkers of inflammation and kidney function. Yet, plasma VEGF-A and PLGF did correlate with blood pressure regulation in the 24h blood pressure profile. Interestingly, increased blood pressure was observed as complication of anti VEGF antibody therapy in patients with macular edema and poorly controlled arterial hypertension [25]. Moreover, a recently published meta-analysis of serious adverse events associated with intravitreal VEGF antibody treatment has confirmed a significantly increased incidence of strokes as a complication linked to post treatment decline of circulating VEGF [3].

As reported earlier, CTAD plasma VEGF-A levels were lower than serum VEGF-A concentrations and might better reflect free circulating VEGF-A [11]. In our exploratory study, VEGF-A serum levels were ~ 34 fold and 22 ~ fold higher than in CTAD plasma of patients with type 2 diabetes and controls, respectively. CTAD is a standard formulation to inactivate platelets, the main source of VEGF-A. Indeed, previous studies showed that CTAD can minimize platelet activation to as low as 1% [18]. In a recent report, EDTA plasma was compared to PECT (containing a mixture of anticoagulants to which prostaglandin E_1_ and theophylline was added) and CTAD plasma in 6 healthy volunteers [18]. In line with our results, CTAD plasma VEGF-A levels were lowest, followed by PECT- and EDTA-plasma. Moreover, we and others confirmed earlier that serum VEGF-A levels are largely higher relative to plasma levels in patients with type 1 and 2 diabetes, metabolic vascular syndrome, different malignancies and rheumatoid arthritis [4,8,9,11,23,24,26–29].

Our study has clear limitations. It is an exploratory study in two small cohorts with no reference to quantitative funduscopy such as OCT parameters. Moreover, we failed to match patients for statin use, which has been shown to moderately reduce VEGF-A levels [10]. However, both groups, controls and patients, where comparable in terms of daily and seasonal VEGF fluctuations, which argues against a major effect of statins on the results in our patients. In addition, VEGF-C and PLGF were only determined in plasma. The strength of the study is the well characterized population and the standardized conditions, documented by stable HbA1c, body weight, blood pressure and parameters of inflammation. Thus, we can give a clear answer to our primary objective: VEGF levels are stable over half a year, a period typical for time schedules in anti VEGF treatment. Moreover, our data show that blood samples for VEGF-A can be taken in the fasted or postprandial state after 7.30 AM.; VEGF-C and PLGF should be measured in the fasted state. However, it remains an open question which measurements—serum, EDTA plasma, CTAD plasma or other plasma formulations—confer the best readout of risk associated with VEGF-A.

Studies with parallel measurements of intravitreal VEGF concentrations and in plasma have demonstrated a significant correlation between intravitreal and systemic concentrations [30]. If that is valid for patients with DME, circulating VEGF levels could be used to guide the treatment with VEGF antibodies. Furthermore, prospective studies with OCT and functional measurement of retinal flow and kidney function are urgently needed to quantify the putative risk associated with systemic VEGF-A levels. This should be relevant for time and dosage of treatment of DR with anti VEGF antibodies.

## Supporting information

S1 File CONSORT flow diagram. (DOCX)
