# Supplementary material for: Intra-individual variability and circadian rhythm of vascular endothelial growth factors in subjects with normal glucose tolerance and type 2 diabetes
Source: PLoS One. 2017 Oct 9;12(10):e0184234. doi: 10.1371/journal.pone.0184234 (PMC5633167; doi:10.1371/journal.pone.0184234)
Supplement: S1 File — (DOCX) [file pone.0184234.s001.docx]

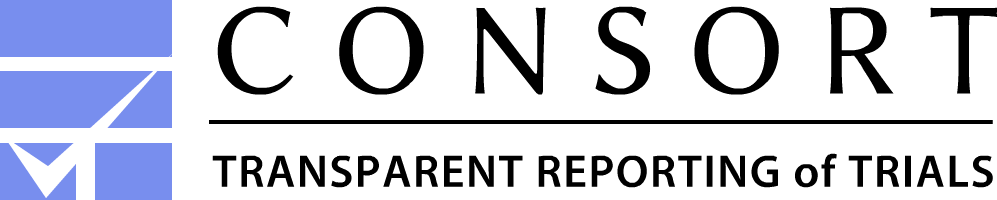


**CONSORT 2010 Flow Diagram**

No intervention

## Enrollment

Randomized (n= 40)

Excluded (n=3 )

♦  Not meeting inclusion criteria (n=2)

♦  Declined to participate (n=1 )

♦  Other reasons (n= 0)

Assessed for eligibility (n= 43)
